# Factors associated with mortality in pediatric pneumonia patients supported with mechanical ventilation in developing country

**DOI:** 10.1016/j.heliyon.2021.e07063

**Published:** 2021-05-18

**Authors:** Ari Meliyanti, Desy Rusmawatiningtyas, Firdian Makrufardi, Eggi Arguni

**Affiliations:** Department of Child Health, Faculty of Medicine, Public Health and Nursing, Universitas Gadjah Mada/Dr. Sardjito Hospital, Yogyakarta, Indonesia

**Keywords:** Mortality, Pneumonia, Ventilator, Bacteremia, Underweight

## Abstract

**Background:**

Pneumonia is still a major cause of death and incurs significant morbidity and mortality in developing countries. Thus, patients care does not only focus on treatment but also identifying factors that associated with the patient's outcome. Therefore we defined factors associated with mortality in pediatric pneumonia and assessed the outcome of pneumonia supported by mechanical ventilation in children.

**Methods:**

We performed cohort retrospective study by collecting data of pediatric pneumonia patients who admitted to Pediatric Intensive Care Unit (PICU) at Dr. Sardjito General Hospital, from 2014 to 2016. Chi square and multivariate logistic regression tests were used to analyze the variables: anemia, comorbidities, bacteremia, age between 1-6 months old, and underweight as associated factors for mortality.

**Results:**

One hundred and eleven children were included in this study. Those patients were diagnosed as community acquired pneumonia (79.3%), hospital acquired pneumonia (14.4%) and ventilator associated pneumonia (6.3%), with mortality rate 47.7%. Multivariate logistic regression analysis revealed that bacteremia, and underweight could be used as predictor factors of mortality for pediatric patients with pneumonia who were supported by mechanical ventilation with OR 2.5 (CI 95%: 1.03–6.1) and 2.4 (CI 95%: 1.1–5.7), respectively.

**Conclusion:**

Factors associated with mortality for pediatric patients with pneumonia who were supported by mechanical ventilation were bacteremia and underweight. It is necessary to compare our findings with other centers.

## 1 Introduction

Childhood pneumonia is still a major cause of death and incurs significant morbidity and mortality in developing countries [1]. Incidence of pneumonia in developed countries is estimated to be at 33 per 10,000 for children under 5 years old and 14.5 per 10,000 for children up to 16 years old [2]. Mortality rate of pneumonia is lower in developed countries, being <1 per 1000 per year [3]. In Indonesia, pneumonia's prevalence in general population has increased from 2.1% in year 2007 to 4.5 % in year 2013, with an incidence rate of 1.8%. Pneumonia's period prevalence based on age group shows that pneumonia happens most frequently in the 1–4 years old age group. Mortality rate is higher in infants with 2.89% compared to 0.2 % in children aged 1–4 years old [4, 5].

For the last 2 decades, technological advancement in life supporting equipment has caused an increase in the number of intensive care facilities in hospitals [6]. Mechanical ventilation is a widely used form of respiratory support in pediatric intensive care units (PICU). However, prolonged use of mechanical ventilation causes complications and leads to mortality [7,8]. A study revealed that children less than 5 years old were most vulnerable to major diseases and the majority of them are treated in the PICU. Worldwide statistics in all age groups indicate pneumonia remains the main cause of PICU utilization, and is also associated with a mortality rate of 29.9% [9].

There were only a few studies about mortality predictive factors for pneumonia patients in the PICU that had been done. A study in 2017 concluded that hospital acquired pneumonia (HAP) and bacteremia were risk factors for mortality in pneumonia, with OR 2.92 and 5.03, respectively [10]. In 2012, a study revealed that congenital heart disease [11], Down syndrome, and cerebral palsy served as risk factors for fatality of severe pneumonia in PICU [12]. Immunodeficiency was previously reported as independently predictor of mortality in pediatric pneumonia (OR 14; 95% CI: 8.2–23) [13]. Another study in the PICU reported that septic shock, severe acute respiratory distress syndrome, neutropenic sepsis and chronic liver disease were independent predictor factors of mortality for patients with severe pneumonia [14]. Several studies reported the mortality of pneumonia patients in the tertiary hospital PICU, but none of them correlated it with factors associated mortality in Indonesia. Here, we estimated that several factors associated with mortality in PICU patients. This study is aimed to evaluate the outcome of patients with pneumonia admitted to PICU and its associated factors.

## 2 Material and methods

### 2.1 Study design and population

We did cohort retrospective study by collecting patients data from 2014 to 2016 who had been diagnosed with pneumonia and admitted to PICU in our tertiary hospital (Dr. Sardjito General Hospital, Yogyakarta, Indonesia). We used pneumonia criteria based on the World Health Organization Guideline 2005 [15]. All patients were follow up until they reached PICU outcome as “survive” and “death”. Included patients were children who were admitted to PICU with pneumonia. We excluded patients with incomplete data for establishing penumonia definition and other predictor data on their medical record.

### 2.2 Data collection

We recorded data from all patients during their PICU admission. The data were gender, age, nutritional status, chronic disease comorbidity, whether they are referred from other hospital, and other PICU. Potential predictors included epidemiological and clinical variables. The following data were evaluated as potential factors associated with mortality: bacteremia, anemia, age less than 6 months old, underweight and comorbidity.

All data were obtained from medical records during hospitalization. We defined age as the patient's age when being diagnosed with pneumonia at PICU. Nutritional status was classified using the WHO growth chart: Weight-for-Height curve for children younger than 5 years old or BMI-for-age curve for children 5 years old or older. Children were categorized as having good nutritional status (-2 < z < 2 SD), being under nourished (z < -2 SD) and overweight (z > 2 SD) [16]. Comorbidity were defined as any chronic condition that has been diagnosed and treated before the patient was diagnosed with pneumonia, in this study we recorded congenital heart disease, neurocognitive problems, down syndrome, immunodeficiency and lung congenital abnormality.

We defined anemia based on laboratoric results and recommendation of WHO anemia diagnosis in 2011. Children were categorized as having anemia and non-anemia based on age of children [17]. We defined bacteremia as the positive growth of blood culture taken at the first time of pneumonia diagnosis established after assessing the isolate was a true pathogen [18].

### 2.3 Outcome measures

The follow-up period ended when every patient reached their PICU outcome, which was defined as survive or death. The mortality rate of pneumonia was defined as the proportion of patients who developed pneumonia and died during hospitalization among children admitted to PICU at the study period [19].

### 2.4 Data analysis

Data were analyzed using IBM SPSS Statistics 23rd version (IBM Corp., Chicago). We estimated power of this study was 0.84, this result was obtained by comparing the proportion of two independent samples. The significance for predictor was assessed using the Chi-squared test and p < 0.05 was considered to indicate statistical significance. We performed multivariate logistic regression to get Odds ratio (OR) with 95% Confidence Interval (CI) to evaluate factors associated with mortality. We also permormed Hosmer-Lemeshow test and found 0.417 for this study.

### 2.5 Ethics approval

This study approved without individual patients' consents needed by the Ethics Committee of the Faculty of Medicine, Universitas Gadjah Mada, Yogyakarta, Indonesia KE/FK/0860/EC/2019.

## 3 Results

Characteristics of subjects examined in this study consisting of gender, nutritional status, anemia, comorbidity, length of stay in PICU, duration of ventilator used, number of patients diagnosed with community acquired pneumonia (CAP), hospital acquired pneumonia (HAP), and ventilator acquired pneumonia (VAP) are shown in Table 1. Most of the patients were diagnosed with CAP (79.3%) while 14.4% were diagnosed with HAP and 6.3% were diagnosed with VAP. Out of the 111 patients that fulfilled the inclusion criteria, 53 (47.7%) died in the PICU. Median length of stay in PICU was 9 days with interquartile range (IQR) of 12 days. Median of ventilator use duration was 7 days with interquartile range (IQR) of 10 days.

**Table 1:** Subjects characteristics.

Variables	Death	Survive	OR	*p*
Gender, n (%)				
Boys	22 (42%)	32 (55%)	0.58	0.15
Girls	31 (58%)	26 (45%)	1.14	0.72
Nutritional status, n (%)				
Underweight	34 (64%)	27 (46%)	2.1	0.06
Normal	19 (36%)	31 (53%)	0.49	0.06
Anemia, n (%)				
Yes	39 (74%)	39 (67%)	1.4	0.46
No	14 (26%)	19 (33%)	0.17	0.01
Comorbids, n (%)				
Yes	35 (66%)	34 (59%)	1.4	0.40
No	18 (34%)	24 (41%)	0.73	0.42
PICU duration (days), median (min-max)	7 (1–71)	10 (3–57)	0.51	0.21
Median ventilator usage (days), (min-max)	6 (1–71)	7 (1–51)	0.58	0.36
*Community acquired pneumonia* (CAP), n (%)	40 (75%)	48 (83%)	0.64	0.35
*Hospital acquired pneumonia* (HAP), n (%)	10 (19%)	6 (10%)	2.02	0.21
*Ventilator acquired pneumonia* (VAP), n (%)	3 (6%)	4 (7%)	0.81	0.79

min-max: minimum-maximum value.

Frequency of each comorbidity and patients with the end result of death for each respective comorbidity are illustrated in Table 2.

**Table 2:** Comorbidities.

Comorbid type	Death (n)	Survive (n)	Total (n)
Congenital heart disease	21	10	31
Neurocognitive problems	8	19	27
Down syndrome and congenital heart disease	3	3	6
Immunodeficiency	1	0	1
Lung congenital abnormality	0	1	1

Chi-square analysis for 5 predictor factors and mortality found that 2 variables have a p value of <0.25. The 2 variables that were determined to be statistically significant were underweight (p = 0.06) and bacteremia (p = 0.05) and undergone multivariate analysis. The results of the bivariate analysis are shown in Table 3.

**Table 3:** Bivariate analysis on the predictor factor.

Variable	Death (n)	Survive (n)	OR	*p*
Age 1–6 month	28	30	1.1 (0.5–2.2)	0.90
Age >6 month	25	28	0.96 (0.45–2.02)	0.91
Underweight	34	27	2.1 (0.96–4.4)	0.06∗
Comorbids	35	34	1.4 (0.6–3.0)	0.42
Anemia	39	39	1.4 (0.6–3.1)	0.47
Bacteremia	22	14	2,2 (1.0–5.0)	0.05∗

∗ Statistically significant, *p* < 0.25.

Logistic regression analysis towards the 2 statistically significant variables showed that underweight (p = 0.037), and bacteremia (p = 0.043) were mortality predictor factors for pediatric patients with pneumonia on mechanical ventilators in the PICU. The multivariate analysis results are illustrated in Table 4.

**Table 4:** Multivariate analysis on predictor factors of mortality outcome.

Death (n = 53)			
Variables	OR	CI 95%	*p*
Underweight	2.4	1.1–5.7	0.037∗
Bacteremia	2.5	1.03–6.1	0.043∗

∗ Means statistically significant, *p* < 0.05.

## 4 Discussion

During the study period, 111 patients were admitted to the PICU of Dr. Sardjito General Hospital with pneumonia, of whom 53 (48%) were deceased. We found that bacteremia and underweight were associated with mortality.

Our study located in one of the university based referral hospital in Indonesia equipped with tertiary-PICU facility that mostly patients with pneumonia referred from surrounding area. This is among the first studies to investigate the association between epidemiological and clinical characteristics with mortality of children with pneumonia supported with mechanical ventilation who were treated in PICU in Indonesia. The limitation of this study was the retrospective design employed, and that might be an error in recording data. This could happen because we still use manual medical record. We tried to minimize this by adding a second data collector who validated the data input processes. This study is also a single-center study, that might not reflect overall situation of pediatric pneumonia in Indonesia.

Patients with underweight nutritional status in this study were reported to be up to 55%, with a mortality of 30.6%. This finding compare with study in Malawi in the year 2016 also reported a high mortality rate of 34.8% among pneumonia patients with poor nutritional status [20]. Underweight nutritional status is a statistically significant predictor of mortality in this study with a OR 2.4 (CI 95%: 1.1–5.7). In the previous study in 2010, malnourished status was found to be a predictor of mortality in pneumonia patients with an OR of 3.8, although this value was determined to be statistically insignificant (p = 0.25) [7]. This phenomenon might be caused by the reduced amount of T lymphocytes, complements, and bactericidal activity of neutrophils that causes malnourished children to not be able to fend off infection adequately compared to healthy children resulting in more severe infection or death [21].

Bacteremia occurred in 32.4% of our subjects. It was proven by the evidence of patients' blood culture results during treatment in PICU. The mortality rate of patients with bacteremia in our study was 41.5%, which was different from the results of a previous study that reported the incidence rate of bacteremia was 3.3% with 26.6% of mortality rate [22]. The difference in the results may be caused by the differences in the study settings and patients' characteristics. Bacteremia was found to be a significant predictor factor for mortality in our study (OR 2.5; CI 95%: 1.03–6.1). A previous study revealed the same result (OR 5.03; CI95%: 1.77–14.35) [10]. Bacteremia is the presence of pathogenic bacteria in circulation that has been found to have a significant relationship with mortality and morbidity in children [23]. Bacteremia has also been used to predict the presence of pneumonia caused by bacterial infection, which frequently had more severe clinical manifestations and complications [24].

According to our results, the age range of 1–6 months was not a significant mortality predictor (OR 1.05; CI 95%: 0.5–22, with p = 0.9). This result is different from the previous study which reported age less than 6 months as a significant mortality prognostic factor of children with pneumonia (OR 2.2; CI 95%: 1.14–4.2, with p = 0.018) [25]. In younger children, there are many factors including: less number of alveolus, less cartilage number, small diameter of respiratory tract, stiff bronchial smooth muscle, and a suboptimal respiratory tract immune system which can contribute to pneumonia manifesting in more severe clinical manifestations [26].

Comorbid factors did not contribute as significant mortality predictors (OR 1.4; CI 95%: 0.6–3.0, p = 0.5). This finding was different compared to the previous study which reported that grade II comorbids (congenital heart disease, asthma, down syndrome and moderate malnutrition) might contribute as significant mortality prognostic factors in pneumonia patients (OR 4.9; CI 95%: 2.8–8.5, p < 0.01). Grade III comorbids (HIV, immunosuppressive therapy utilizing 2 mg/kgbw/day more than 15 days of therapy, malignancy or severe malnutrition) were also reported as significant mortality predictors (OR 6.18; CI 95%: 3.4–10.9, p = < 0.001) [25]. The discrepancies between these reports to our study may be caused by our attempt to separate nutritional status in the variable evaluation from the comorbid variable.

Anemia may play an important role as a factor associated with mortality in pneumonia (OR 4.4; CI 95%: 1.25–15.5, p = 0.021) [7], however our study failed to prove it (OR 1.4, CI 95%: 0.6–3.1, p = 0.47). To date, hemoglobin has been known to play an important role in transporting oxygen throughout the body and every 1 g of hemoglobin will bind around 1.39 ml/dl blood, thus a lowered level of hemoglobin will result in less oxygen transported [27]. One study in Kenya showed an increase of mortality rate in patients with hemoglobin level less than 5 g/dl [17]. In our study, anemia could not be used as a predictor factor due to the high cut-off value, 11–13 g/dl according to WHO standards, and thus the mortality outcome was lower [28].

It is necessary to perform further studies utilizing a prospective design in order to minimize the risk of bias. However, the main contribution of this study is in filling the gap in empirical research since there has been no studies about factors associated with mortality in pediatric patients with pneumonia supported by mechanical ventilator who were admitted to the PICU which have been published in Indonesia.

## 5 Conclusion

In conclusion, factors associated with mortality for pediatric patients with pneumonia who were supported by mechanical ventilation were bacteremia and underweight. It is necessary to compare our findings with other centers.

## Declarations

### Author contribution statement

Ari Meliyanti: Conceived and designed the experiments; Performed the experiments; Analyzed and interpreted the data; Wrote the paper.

Desy Rusmawatiningtyas, Firdian Makrufardi and Eggi Arguni: Conceived and designed the experiments; Analyzed and interpreted the data; Wrote the paper.

### Funding statement

This research did not receive any specific grant from funding agencies in the public, commercial, or not-for-profit sectors.

### Data availability statement

Data will be made available on request.

### Declaration of interests statement

The authors declare no conflict of interest.

### Additional information

No additional information is available for this paper.
